# Artificial neural networks to predict future bone mineral density and bone loss rate in Japanese postmenopausal women

**DOI:** 10.1186/s13104-017-2910-4

**Published:** 2017-11-10

**Authors:** Mitsunori Shioji, Takehisa Yamamoto, Takeshi Ibata, Takayuki Tsuda, Kazushige Adachi, Noriko Yoshimura

**Affiliations:** 1 Department of Gynecology, Minoh City Hospital, Minoh, 562-8562 Japan; 2 Department of Pediatrics, Minoh City Hospital, Minoh, 562-8562 Japan; 3 Department of Internal Medicine, Minoh City Hospital, Minoh, 562-8562 Japan; 4 Department of Orthopedic Surgery, Minoh City Hospital, Minoh, 562-8562 Japan; 5 Department of Preventive Medicine for Locomotive Organ Disorders, 22nd Century Medical and Research Center, The University of Tokyo, Tokyo, 113-8655 Japan; 6 Present Address: Department of Gynecology, Toyonaka Municipal Hospital, Toyonaka, 560-8565 Japan; 7 Present Address: Department of Orthopedics, Kansai Rosai Hospital, Amagasaki, 660-8511 Japan

## Abstract

**Objective:**

Predictions of the future bone mineral density and bone loss rate are important to tailor medicine for women with osteoporosis, because of the possible presence of personal risk factors affecting the severity of osteoporosis in the future. We investigated whether it was possible to predict bone mineral density and bone loss rate in the future using artificial neural networks.

**Results:**

A total of 135 women over 50 years old residing in T town of Wakayama Prefecture, Japan were analyzed to establish a statistical model. Artificial neural networks models were constructed using the two variables of bone mineral density and bone loss rate. The multiple correlation coefficients between the actual and measured values for lumbar and femoral bone mineral densities in 2003 showed R^2^ = 0.929 and R^2^ = 0.880, respectively, by linear regression analyses, while the values for bone loss rates in lumbar and femoral bone mineral densities were R^2^ = 0.694 and R^2^ = 0.609, respectively. Statistical models by artificial neural networks were superior to those by multiple regression analyses. The prediction of future bone mineral density values estimated by artificial neural networks was considered to be useful as a tool to tailor medicine for the early diagnosis of and intervention for women osteoporosis with women.

**Electronic supplementary material:**

The online version of this article (10.1186/s13104-017-2910-4) contains supplementary material, which is available to authorized users.

## Introduction

Fragility fractures of bones are one of the major problems in the field of postmenopausal osteoporosis. Especially, fractures of the proximal femur (femoral) often have a markedly negative impact on the quality of life of affected patients [1]. In addition, medical care for these patients is expensive [2]. Thus, the early diagnosis and prevention of osteoporosis are important medical issues in an advanced aging society. The diagnosis of osteoporosis is based on the presence of fragile bone fractures or low bone mineral densities (BMD), which are usually measured by dual energy X-ray absorptiometry (DXA). For the latter, values of no more than 70% YAM (young adult mean values), the same as a T-score (the bone density compared with what is normally expected in a healthy young adult of your sex in terms of standard deviations) ≤ − 2.5, were designated as cut-off values for the diagnosis of osteoporosis [3].

Artificial neural networks (ANN) are artificial adaptive systems that simulate certain characteristics of the human brain [4]. They are particularly suited for solving nonlinear problems. The ability to learn in an adaptive way makes ANN models powerful tools for data analysis. ANN has been shown to improve the predictive value of statistics in many areas of medicine [5]. In the field of bone metabolism, several studies reported about the efficacy of ANN. It was reported that the performance of ANN models for the prediction of BMD values using several parameters among postmenopausal women was superior to the conventional regression methods [6]. Also, ANN showed a better performance for predicting morphometric vertebral fractures in postmenopausal osteoporosis than logistic regression analysis [7]. However, no study has investigated the ability of ANN to predict future BMD and bone loss rate (BLR) values among postmenopausal women.

Thus, the aim of the present study was to investigate whether ANN models are able to predict future BMD and BLR values from person to person at lumbar and femoral sites of postmenopausal women, respectively, in the future. This is expected to be useful in combination with the use of FRAX (fracture risk assessment tool), an additional tool for the future prediction of bone fractures [8].

## Main text

We constructed the statistical model of ANN using the information on female participants in Taiji study, T town, Wakayama, Japan. The study was designed as a population-based cohort, and it has been profiled in detail elsewhere [8, 9]. We have reported several studies on osteoporosis based on the data of Taiji study concerning BMD [8], risk factors affecting BMD [9] and determinants of bone loss [10].

In the present study, we used the information of 135 female participants with age ≥ 50 years at the baseline. Profiles of the subjects in the present study were shown in Additional file 1: Table S1. Input variables consisted of eleven variables: age, weight, height, age at menopause, age at menarche, durations after menopause, body mass index (BMI), percent of body fat, fat mass, lean body mass, and lumbar (L2–L4) or femoral BMD values which were measured in 1993, respectively. BMD was measured by DXA (HologicQDR-1000). Percent fat mass values were obtained using a reported formula [11]: body fat percent = 1.20 × BMI + 0.23 × age (years old) − 5.4. Then, the fat and lean body masses were calculated using body weight values. Output variables consisted of two variables: BMD at lumbar site (L2–L4) (LBMD) or BMD at proximal femur site (FBMD) values measured in 2003 and the BLR, respectively, calculated by the difference in BMD values from 1993 to 2003 divided by 10 (Additional file 2: Figure S1a, b). To increase the efficiency of ANN, we used values normalized by the transformations as follows except BMD and BLR values. First, we calculated (the difference between the measured and minimum values) divided by (the difference between the maximal and minimum values) for each input parameter. These maximal and minimal values were taken from the distribution of values from the entire study content. We obtained final values by taking each calculated value previously obtained and multiplying it by 0.8 (0.9–0.1) and then adding 0.1 to normalize the values from 0.1 to 0.9.

To predict BMD and BLR in 10 years at lumbar and femoral sites of postmenopausal women, ANN models were built. In detail, multilayer perceptrons were used with 11 neurons in the input layer, four neurons in one hidden layer, and two neurons in the output layer. After performing a number of trials (around 50–100 times), the most appropriate model was selected based on the better R^2^ value by the single and fivefold cross-validation methods. In addition, we also obtained predicted values for these parameters by multiple regression analyses (MRA).

To evaluate the predicted BMD and BLR values at lumbar and femoral sites, we used linear regression analyses. The statistical comparison of each statistical model was performed using Akaike’s information criterion (AIC), Schwartz’s Bayesian information criterion (BIC), and multiple correlation coefficients (R^2^) values corrected by degrees of freedom (R^2^ values), respectively. For the diagnosis of osteoporosis, we estimated the sensitivity, specificity, and c-index by receiver operating characteristic (ROC) analyses to evaluate the methods of ANN and MRA. Data are shown as a mean with 95% confidential interval (CI) values. We used JMP 8.0 (SAS, Japan) for the analyses of ANN. For the other statistical analyses, we used JMP9.0 or Dr. SPSS II (SPSS), respectively. A P value of < 0.05 was considered significant.

**Fig. 1:**
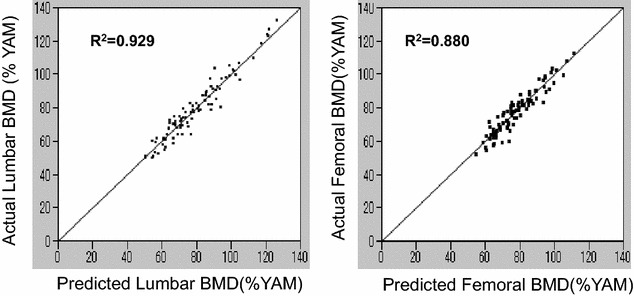
Correlations between the actual and predicted lumbar and femoral BMD values by artificial neural networks. The horizontal and vertical lines show the predicted and actual BMD values, respectively. R^2^ values were calculated by linear regression analyses

**Fig. 2:**
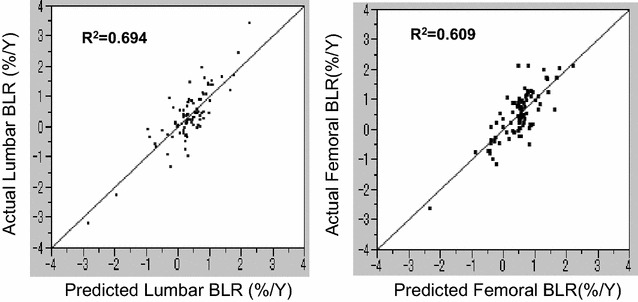
Correlations between the actual and predicted lumbar and femoral BLR values by artificial neural networks. The horizontal and vertical lines show the predicted and actual BLR values, respectively. R^2^ values were calculated by linear regression analyses

The R^2^ values between the actual and predicted values of LBMD and FBMD in 2003 were revealed to be R^2^ = 0.929 and R^2^ = 0.880, respectively, by linear regression analyses (Fig. 1), while the values for BLR of LBMD (LBLR) and FBMD (FBLR) were 0.694 and 0.609, respectively (Fig. 2). The sensitivity, specificity, and c-index for the predicted diagnosis of osteoporosis for the LBMD and FBMD in 10 years using this model were 80.0, 90.5%, and 0.825 and 80.6, 93.3%, and 0.870, respectively (Table 1).

**Table 1:** Comparison of statistical models

	AIC	BIC	R^2^	Sensitivity (%)	Specificity (%)	c-index
Lumbar BMD						
ANN	551	558	0.929	80.0	90.5	0.825
MRA	636	643	0.803	83.3	87.3	0.853
Femoral BMD						
ANN	538	545	0.880	80.6	93.3	0.870
MRA	625	632	0.687	61.3	93.3	0.773
Lumbar BLR						
ANN	131	139	0.694			
MRA	228	325	0.137			
Femoral BLR						
ANN	135	143	0.609			
MRA	206	213	0.153			

We also compared the statistical models obtained by ANN with those by MRA for LBMD and FBMD values, respectively. For both sites of bone, AIC and BIC values for BMD by ANN were much lower than by MRA (Table 1), suggesting that the statistical models obtained by ANN were superior to those by MRA. Also, R^2^ values obtained by ANN for lumbar and femoral sites (0.929 and 0.880, respectively) were much higher than those by MRA (0.803 and 0.687, respectively). In addition, R^2^ values obtained by ANN for LBLR and FBLR (0.694 and 0.609, respectively) were much higher than those by MRA (0.137 and 0.153, respectively). These data re-confirmed that the statistical method of ANN was superior to that of MRA (Table 1). Moreover, we further compared the statistical models by ANN for LBLR and FBLR with those for LBMD and FBMD, respectively (Table 1). The R^2^ values for LBLR and FBLR were 0.694 and 0.609, respectively, being much lower than those for LBMD (0.929) and FBMD (0.880), suggesting that the statistical models for BLR were inferior to those for BMD (Table 1).

We showed that we could predict future BMD and BLR in 10 years on person-to -person basis using conventional parameters. These parameters were the age, weight, height, age at menopause, age at menarche, durations after menopause, BMI, percent of body fat, fat mass, lean body mass, and lumbar (L2–L4) or femoral BMD values. Because they were easy to obtain on the first visit to osteoporosis clinics, the use of these findings may further reduce future bone fractures of post-menopausal women.

Reducing the bone fracture rate of women is one of the goals of therapy for osteoporosis. Since the bone mineral density is responsible for 70% of the bone strength and because the occurrence of bone fractures is thought to be associated with the bone strength [12], the discovery of a new tool in this study to predict future BMD values may be useful to reduce the bone fracture rate in post-osteoporosis women. In addition, bone fractures of the proximal femur often impair the quality of life of affected patients, with increases in medical care and costs. Thus, the early diagnosis and prevention of osteoporosis are important medical issues in an advanced aging society. Because of the possible presence of different personal risks for the severity of osteoporosis in the future, our findings showing the ability to predict individual BMD in 10 years are thought to be useful as a tool of tailored medicine, which might contribute to some extent to the prevention of and decisions regarding early therapy for post-menopausal osteoporosis.

FRAX is the only tool to predict future bone fracture in 10 years. FRAX is thought to be a tool reflecting all fractures derived from secondary osteoporosis, such as rheumatoid arthritis, steroid osteoporosis, type I diabetes mellitus, and hyperthyroidism [13]. On the other hand, our data were based on the only data for women with post-menopausal osteoporosis. Thus, the combined use of FRAX and the detailed analysis by using ANN models may lead to increased power for the future prediction of bone fractures due to post-menopausal osteoporosis.

ANN are artificial adaptive systems that emulate certain characteristics of the human brain [1]. The advantages of ANN include their ability to extract hidden features from input information and their robustness against assumptions concerning the type of distribution of input data and against the influence of diagnostic noise. In many reports, ANN have been used to predict the present BMD based on risk factors for osteoporosis. Certain factors such as the age, a low body weight, and years since menopause have been suggested by most studies to be associated with a low bone mass [14, 15]. However, the prediction of a future BMD using ANN has not been reported. In this study, R^2^ values between the actual and predicted values for of LBMD and FBMD were 0.929 and 0.880, respectively, based on linear regression analyses. In addition, the sensitivity, specificity, and c-index for the predicted diagnosis of osteoporosis of the LBMD in 10 years using this model were 80.0, 90.5%, and 0.825 while the same values for FBMD were 80.6, 93.3%, and 0.870, respectively. We suggest that our method is applicable for clinical use for the prevention of and decisions regarding early therapy for postmenopausal osteoporosis. To our knowledge, this is the first study to show the usefulness of ANN for the prediction of BMD at lumbar and femoral sites in the future.

We also showed in our study that ANN were superior to MRA for predicting future LBLR and FBLR values, respectively. These data are expected because it is very well known that the prediction capability of ANN is superior to MRA. However, this is the first investigation suggesting that ANN are clearly superior to MRA for predicting LBLR and FBLR in future. We speculated that the superiority of ANN might be derived from the fact that some of the biological findings might be non-linear in nature, although the actual reasons remain unknown.

It is concluded that the application of ANN to predict future BMD in advance of the first visit of a patient to an osteoporosis clinic may lead to early intervention to avoid possible fragile bone fractures due to severe post-menopausal osteoporosis.

## Limitations

Firstly, it is possible that the prediction in our study is only applicable to the BMD of women with osteoporosis living in regions similar to T town, Wakayama, Japan. This is because of the nature of ANN, which processed the data so precisely that the results might only reflect the characteristics of the region where the study was conducted. Since T town is a rural region of Japan, it is possible that our data are not applicable to women with osteoporosis living in big cities with large populations in Japan.

Secondly, the presence of some problems due to the small sample size and the lack of the important input variables such as the daily physical activities, which are thought to have deep impacts on BMD and BLR values via the applied force to bone, need to be investigated by a large cohort from different countries in future.

## Additional files

**Additional file 1: Table S1.** Patient profiles. Data for age, height, body weight, BMI, age at menarche, age at menopause, duration after menopause, percent body fat, lean body mass, fat mass, lumbar BMD in 1993, annual lumbar bone loss rate, femoral BMD in 1993, annual femoral bone loss rate were shown.

**Additional file 2: Figure S1.** a Artificial neural networks for lumbar BMD and lumbar BLR. Input layers consisted of age, weight, height, age at menopause, age at menarche, durations after menopause, BMI, percent of body fat, fat mass, lean body mass, and lumbar (L2–L4) BMD values. Output layers consisted of lumbar BMD in 2003 and lumbar BLR from 1993 to 2003. In hidden layers, we set 4 neurons. All the input variables other than the lumbar BMD in 1993 were normalized. b Artificial neural networks for femoral BMD and femoral BLR. In Figure S1b, we used the left femoral BMD values in the input layers instead of lumbar (L2–L4) BMD values. Output layers consisted of the left femoral BMD in 2003 and femoral BLR from 1993 to 2003. All the input variables other than the femoral BMD in 1993 were normalized.

## Abbreviations

BMD: bone mineral density
BLR: bone loss rate
ANN: artificial neural networks
LBMD: BMD at lumbar site (L2–L4)
FBMD: BMD at proximal femur site (FBMD)
DXA: dual energy X-ray absorptionmetry
YAM: young adult mean values
FRAX: fracture risk assessment tool
MRA: multiple regression analysis
CI: confidential interval
R^2^: multiple correlation coefficients
AIC: Akaike’s information criterion
BIC: Schwartz’s Bayesian information criterion
ROC: receiver operating characteristic
