# Supplementary material for: Artificial neural networks to predict future bone mineral density and bone loss rate in Japanese postmenopausal women
Source: BMC Res Notes. 2017 Nov 10;10:590. doi: 10.1186/s13104-017-2910-4 (PMC5681768; doi:10.1186/s13104-017-2910-4)
Supplement: Supplementary file 1 — Additional file 1: Table S1. Patient profiles. Data for age, height, body weight, BMI, age at menarche, age at menopause, duration after menopause, percent body fat, lean body mass, fat mass, lumbar BMD in 1993, annual lumbar bone loss rate, femoral BMD in 1993, annual femoral bone loss rate were shown. [file 13104_2017_2910_MOESM1_ESM.ppt]

## Slide 1
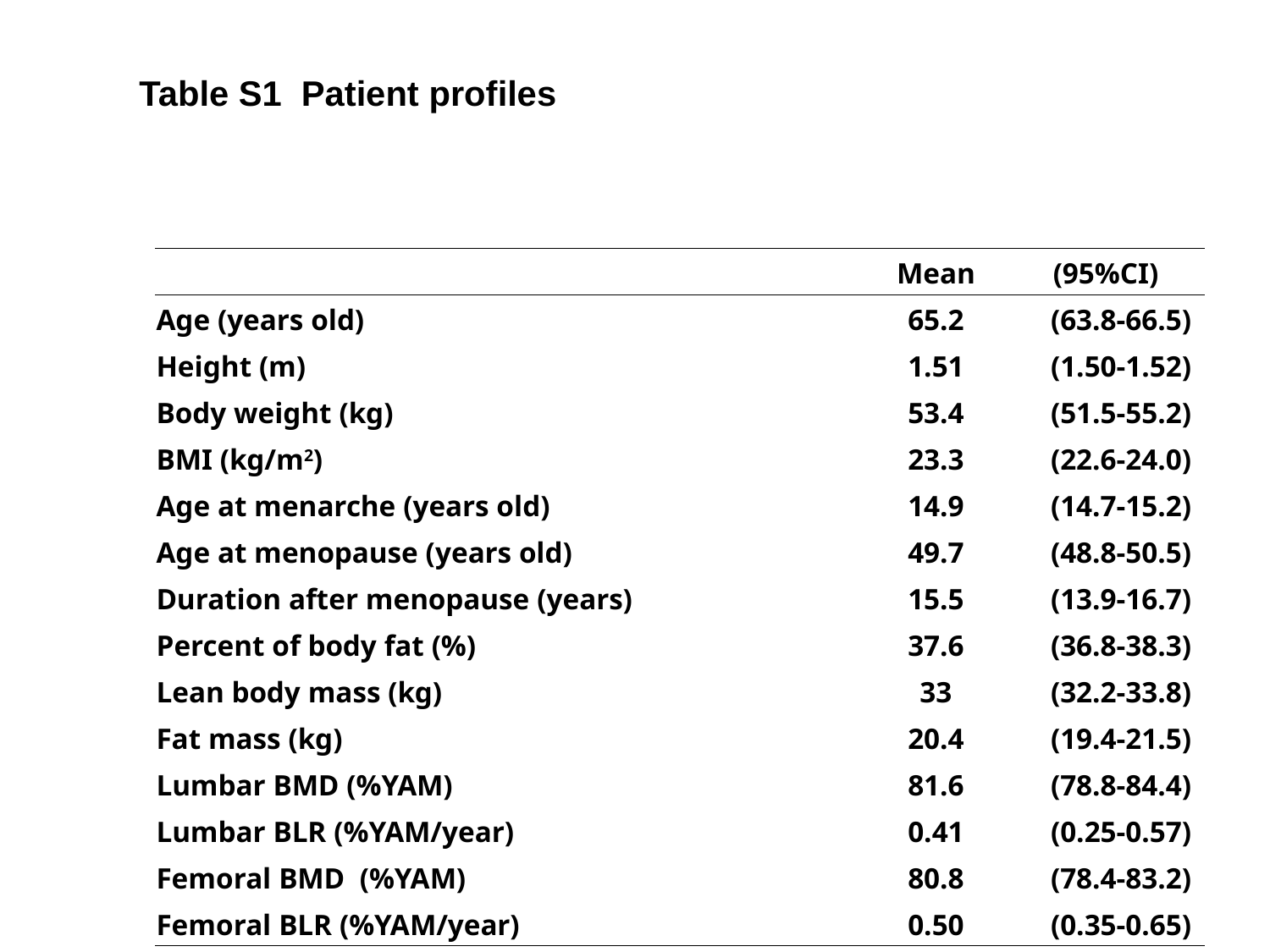

Table S1 Patient profiles
| | | |
| --- | --- | --- |
| | | |
| | Mean | (95%CI) |
| Age (years old) | 65.2 | (63.8-66.5) |
| Height (m) | 1.51 | (1.50-1.52) |
| Body weight (kg) | 53.4 | (51.5-55.2) |
| BMI (kg/m2) | 23.3 | (22.6-24.0) |
| Age at menarche (years old) | 14.9 | (14.7-15.2) |
| Age at menopause (years old) | 49.7 | (48.8-50.5) |
| Duration after menopause (years) | 15.5 | (13.9-16.7) |
| Percent of body fat (%) | 37.6 | (36.8-38.3) |
| Lean body mass (kg) | 33 | (32.2-33.8) |
| Fat mass (kg) | 20.4 | (19.4-21.5) |
| Lumbar BMD (%YAM) | 81.6 | (78.8-84.4) |
| Lumbar BLR (%YAM/year) | 0.41 | (0.25-0.57) |
| Femoral BMD (%YAM) | 80.8 | (78.4-83.2) |
| Femoral BLR (%YAM/year) | 0.50 | (0.35-0.65) |
