# Supplementary figures and images for: Artificial neural networks to predict future bone mineral density and bone loss rate in Japanese postmenopausal women
Source: BMC Res Notes. 2017 Nov 10;10:590. doi: 10.1186/s13104-017-2910-4 (PMC5681768; doi:10.1186/s13104-017-2910-4)

## Slide 1
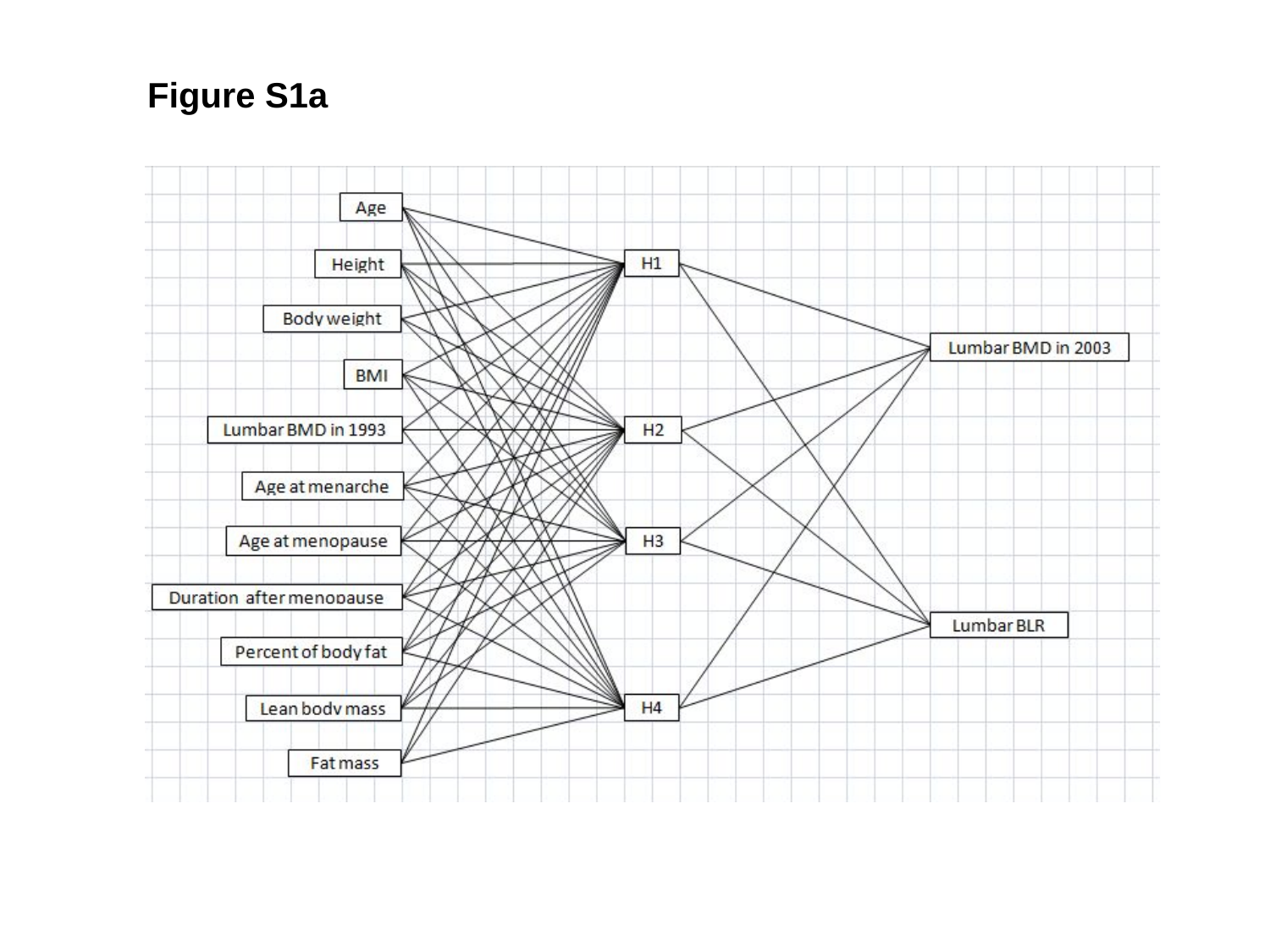

Figure S1a

## Slide 2
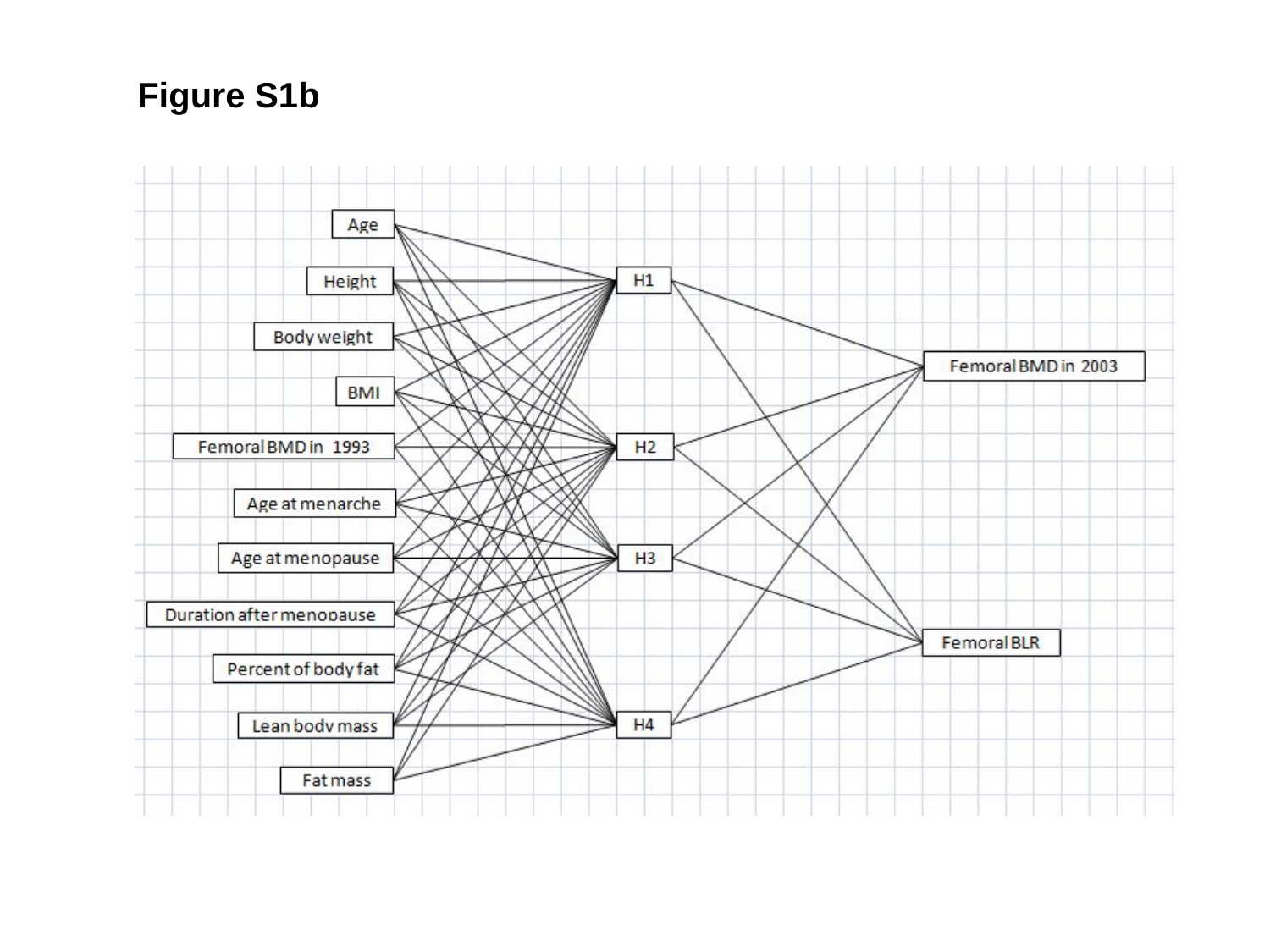

Figure S1b

Supplement: Supplementary file 2 — Additional file 2: Figure S1. a Artificial neural networks for lumbar BMD and lumbar BLR. Input layers consisted of age, weight, height, age at menopause, age at menarche, durations after menopause, BMI, percent of body fat, fat mass, lean body mass, and lumbar (L2–L4) BMD values. Output layers consisted of lumbar BMD in 2003 and lumbar BLR from 1993 to 2003. In hidden layers, we set 4 neurons. All the input variables other than the lumbar BMD in 1993 were normalized. b Artificial neural networks for femoral BMD and femoral BLR. In Figure S1b, we used the left femoral BMD values in the input layers instead of lumbar (L2–L4) BMD values. Output layers consisted of the left femoral BMD in 2003 and femoral BLR from 1993 to 2003. All the input variables other than the femoral BMD in 1993 were normalized. [file 13104_2017_2910_MOESM2_ESM.ppt]
